# Electrochemical Characterization of Graphene and MWCNT Screen-Printed Electrodes Modified with AuNPs for Laccase Biosensor Development

**DOI:** 10.3390/nano5041995

**Published:** 2015-11-20

**Authors:** Gabriele Favero, Giovanni Fusco, Franco Mazzei, Federico Tasca, Riccarda Antiochia

**Affiliations:** 1 Department of Chemistry and Drug Technologies, Sapienza University of Rome, P.le Aldo Moro 5, 00185 Roma, Italy; E-Mails: gabriele.favero@uniroma1.it (G.F.); giovanni.fusco@uniroma1.it (G.F.); franco.mazzei@uniroma1.it (F.M.); 2 Department of Chemistry, Sapienza University of Rome, P.le Aldo Moro 5, 00185 Roma, Italy; 3 Department of Chemistry of Materials, University of Santiago of Chile, Av. Libertador Bernardo O’ Higgins 3363 Estacíon Central, Santiago, Chile

**Keywords:** graphene, multi-walled carbon nanotubes, screen-printed electrodes, gold nanoparticles, laccase, biosensor

## Abstract

The aim of this work is to show how the integration of gold nanoparticles (AuNPs) into multi-wall-carbon-nanotubes (MWCNTs) based screen-printed electrodes and into graphene-based screen-printed electrodes (GPHs) could represent a potential way to further enhance the electrochemical properties of those electrodes based on nanoparticles. Laccase from *Trametes versicolor* (TvL) was immobilized over MWCNTs and GPH previously modified with AuNPs (of 5 and 10 nm). The characterization of the modified electrode surface has been carried out by cyclic voltammetry. The results showed that the use of AuNPs for modification of both graphene and MWCNTs screen-printed electrode surfaces would increase the electrochemical performances of the electrodes. MWCNTs showed better results than GPH in terms of higher electroactive area formation after modification with AuNPs. The two modified nanostructured electrodes were successively proven to efficiently immobilize the TvL; the electrochemical sensing properties of the GPH- and MWCNT-based AuNPs-TvL biosensors were investigated by choosing 2,2′-Azino-bis(3-ethylbenzothiazoline-6-sulfonic-acid diammonium salt (ABTS), catechol and caffeic acid as laccase mediators; and the kinetic parameters of the laccase biosensor were carefully evaluated.

## 1. Introduction

The most important aspect in the construction of biosensors is the orientation and immobilization of the enzyme onto the electrode surface in order to guarantee the highest heterogeneous electron transfer (HET) efficiency between the catalyst and the electrode without losing the catalytic properties of the enzyme. To this end, great interest has been focused on the construction of biosensors based on nanomaterials. Carbon nanotubes (CNTs) have been extensively used as support for sensors [1,2,3,4,5,6] for the unique properties of this material, such as the high surface area ratio and the excellent biocompatibility [7,8,9,10]. On the other hand, graphene (GPH) shows similar electrochemical properties to CNTs, with the huge advantage over the last that does not contain heterogeneous materials [11,12,13,14]. The integration of gold nanoparticles (AuNPs) with MWCNTs or with GPH could represent a potential way to further enhance the electrochemical properties of those nanomaterials [15]. In fact, AuNPs provide a suitable environment for enzyme immobilization, thus facilitating the electron transfer [16,17].

The construction of biosensors based on screen-printed electrodes (SPEs) shows some advantages over conventional electrodes, as they are suitable for working with micro volumes and allow the development of portable, accurate and reproducible sensors for direct *in situ* analysis.

The immobilization of laccase on screen-printed electrodes has been reported in several works [1,18,19]. Laccase enzymes (Lcs, EC 1.10.3.2, p-benzenediol: oxygen oxidoreductase) belong to the family of the multi-copper oxidases (MCOs) [20,21]. Most of the laccases have been extensively studied and, also, have been used to develop high performance biosensors [22,23,24,25,26,27,28].

The typical substrates at the T1 site of Lcs are a variety of organic and inorganic molecules including mono-, di-, and polyphenols, methoxy phenols, aromatic amines and ascorbate [29,30]. Lcs have been widely used for the development of bioelectrochemical devices and in particular biofuel cells [31,32]. Despite the poor substrate specificity, Lcs, have been envisaged as catalysts for the construction of biosensors for phenolic compounds detection [33]. Lcs based biosensors show two advantages: (i) molecular oxygen, the reoxidizing agent, is always present in solution, hence it does not need to be added and (ii) the ability of Lcs to connect directly through direct electron transfer (DET) to the transducers [34], thus allowing the construction of mediator-free electrodes. This last characteristic of Lcs ensures a cheaper design for the biosensor, the lack of heavy metal mediators and the possibility of operating at potentials closer to that of the substrate. Moreover, many MCOs are commercially available.

For those reasons we chose Lcs as model enzyme for our study where we investigated the possibility of entrapping AuNPs with a diameter of 5 nm and of 10 nm onto a support of GPH screen-printed electrodes and MWCNTs screen-printed electrodes to further enhance the HET between Lcs and the electrode support. We performed the immobilization of the AuNPS using a photo-cross-linkable poly(vinylalcohol) (PVA-SbQ) which has been used also for the subsequent immobilization of the enzyme. PVA-SbQ acts as a cross-linking agent [35] improving the immobilization of the AuNPs onto the GHP and onto the MWCNTs screen-printed electrodes, and then favoring the immobilization of Lcs and the stability and durability of the biosensor. Firstly, we tested the electrochemical properties of GPH and MWCNTs screen-printed electrodes before and after the modification with AuNPs and, subsequently, the immobilization efficiency of Lcs onto the AuNPs, which are supported onto the modified GHP and MWCNTs screen-printed electrodes. The studies are performed to evaluate the kinetic parameters for the oxidation of the ABTS, catechol and caffeic acid redox probes.

## 2. Results and Discussion

The electrochemical behavior of GPH and MWCNTs screen-printed electrodes was studied before and after modification with AuNPs in order to assess the ability of the Au-nanomaterial to amplify the electrochemical signal. AuNPs of 5 nm and 10 nm diameter at three different concentrations were used to modify the GPH and the MWCNTs electrodes. The analyses were carried out using potassium ferricyanide as a redox probe.

**Figure 1:**
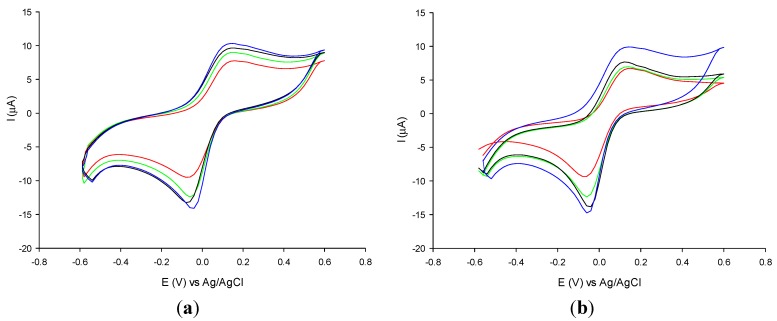
Cyclic voltammograms of 0.1 mM potassium ferricyanide solution at unmodified GPH (red curve) and at GPH modified with AuNPs of 5 nm diameter (**a**) and of 10 nm (**b**) at the following concentrations: 7 × 10^−4^ M (green curve), 1 × 10^−3^ M (black curve), 1.4 × 10^−3^ mM (blue curve). Experimental conditions: 0.1 M phosphate buffer pH 7; scan rate 5 m·Vs^−1^.

Figure 1a,b show the cyclic voltammograms obtained in a solution of potassium ferricyanide with the GPH electrode unmodified (red curves) and modified with 5 nm (Figure 1a) and 10 nm (Figure 1b) AuNPs at different concentrations (curves green, black and blue), respectively. It is possible to observe an increase of the peak currents at increasing AuNPs concentrations for both AuNPs diameters up to 1.4 × 10^−3^ mM AuNPs concentration. Any further increase of the concentration of AuNPs did not show any significant increase of the peak current. The increment corresponds to an increase of the electroactive surface area. The redox probe shows a quasi-reversible behaviour for both unmodified and modified electrodes, with an almost constant Δ*E*p equal to 170 mV, and a ratio between the intensities of the anodic and cathodic peaks close to one. Table 1 reports the electroactive area values of the electrodes calculated by the integration of the anodic peak current and the corresponding roughness factors (ρ). ρ is calculated from the ratio between the effective electroactive area of the modified electrodes and the geometrical area of the unmodified electrodes (3.14 mm^2^). It can be noticed that an increase of the AuNPs concentration generates an increase of the electroactive area and also an increase of the roughness factor (ρ). The study clearly demonstrates an improvement of the electrochemical performances at increasing amounts of AuNPs immobilized onto the electrode surface.

If we compare the electrochemical performances due to the different AuNPs diameter at the same concentration (Figure 1a,b), it is interesting to note that the best performances were obtained with the electrodes modified with AuNPs with the smallest diameter (5 nm). This result might be ascribed to the logical consequence that the particles with the smallest diameter have a higher surface/volume ratio. Nevertheless, other factors could affect the measurements. For example, the photopolymer used for the immobilization of the AuNPs could have allowed a better arrangement of the smallest particles in the polymer network than larger particles, which could have as a consequence a loss of their peculiar characteristics (larger available surface, quantum effect, higher conductivity) in the entrapping process.

**Table 1:** Values of the electroactive area and roughness factor of the unmodified and modified graphene-based screen-printed electrode (GPH) with 5 nm and 10 nm diameter gold nanoparticles (AuNPs) at different concentrations.

Electrode	Electroactive Area (mm^2^)	Roughness Factor (ρ)
GPH	5.0 ± 0.4	1.6 ± 0.2
GPH-AuNPs (5 nm, 7 × 10^−4^ mM)	5.7 ± 0.3	1.8 ± 0.3
GPH-AuNPs (5 nm, 1 × 10^−3^ mM)	6.0 ± 0.4	1.9 ± 0.3
GPH-AuNPs (5 nm, 1.4 × 10^−3^ mM)	6.4 ± 0.3	2.0 ± 0.4
GPH-AuNPs (10 nm, 7 × 10^−4^ mM)	5.5 ± 0.4	1.7 ± 0.2
GPH-AuNPs (10 nm, 1 × 10^−3^ mM)	5.9 ± 0.4	1.9 ± 0.4
GPH-AuNPs (10 nm, 1.4 × 10^−3^ mM)	6.2 ± 0.3	1.9 ± 0.3

**Figure 2:**
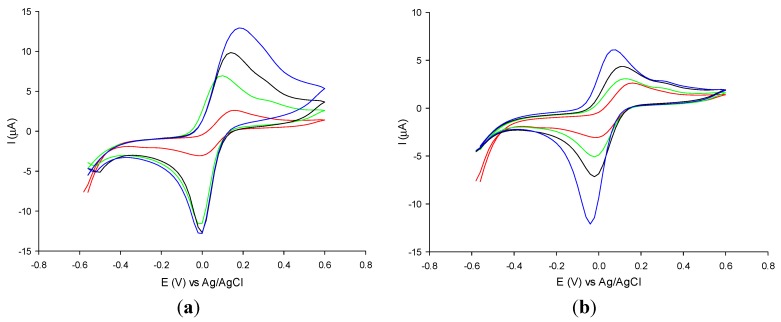
Cyclic voltammograms of 0.1 mM potassium ferricyanide solution at unmodified multi-wall-carbon-nanotubes (MWCNTs) (red curve) and at MWCNTs modified with AuNPs of 5 nm diameter (Figure 2a) and of 10 nm (Figure 2b) at the following concentrations: 7 × 10^−4^ M (green curve), 1 × 10^−3^ M (black curve), 1.4 × 10^−3^ mM (blue curve). Experimental conditions: 0.1 M phosphate buffer pH 7; scan rate 5 m·Vs^−1^.

Similar studies have been carried out with MWCNT electrodes modified with AuNPs of different diameters and at different concentrations. Figure 2a,b show the cyclic voltammograms of potassium ferricyanide obtained with the MWCNT-modified electrode with and without 5 nm and 10 nm diameter AuNPs, at different concentrations. Once again, for both AuNP diameters, the electrodes showed a marked increase of the peak current values with increasing concentrations of AuNPs, as clearly shown in Table 2 where the electroactive area values are reported. For AuNP concentrations higher than 1.4 × 10^−3^ mM AuNPs no significant increase of the peak current has been registered. As for GPH electrodes, MWCNTs electrodes modified with AuNPs with a diameter of 5 nm showed better results in terms of electroactive area and roughness factor.

By comparing the results reported in Table 1 and Table 2 it is possible to note that the MWCNTs electrodes after AuNP modification showed definitely better results in terms of electroactive area compared to GPH electrodes for all AuNP diameters and concentrations used. In particular, by comparing the results obtained with GPH and MWCNTs electrodes modified with AuNPs with 5 nm diameter at the highest concentration which displayed the best electrochemical performances for both nanomaterials employed, the electroactive area of the MWCNTs electrodes was found to be 10% larger than that obtained with the GPH electrodes.

Following those results, TvL was immobilized onto either GPH or MWCNTs modified electrode with the largest employed concentration of AuNPs.

**Table 2:** Values of the electroactive area and roughness factor of the unmodified and modified MWCNTs electrodes with 5 nm and 10 nm diameter AuNPs at different concentrations.

Electrode	Electroactive Area (mm^2^)	Roughness Factor (ρ)
MWCNTs	5.2 ± 0.4	1.7 ± 0.2
MWCNTs-AuNPs (5 nm, 7 × 10^−4^ mM)	6.8 ± 0.3	2.2 ± 0.3
MWCNTs-AuNPs (5 nm, 1 × 10^−3^ mM)	6.9 ± 0.4	2.2 ± 0.4
MWCNTs-AuNPs (5 nm, 1.4 × 10^−3^ mM)	7.1 ± 0.3	2.3 ± 0.3
MWCNTs-AuNPs (10 nm, 7 × 10^−4^ mM)	5.7 ± 0.4	1.8 ± 0.2
MWCNTs-AuNPs (10 nm, 1 × 10^−3^ mM)	6.1 ± 0.4	1.9 ± 0.4
MWCNTs-AuNPs (10 nm, 1.4 × 10^−3^ mM)	6.7 ± 0.3	2.1 ± 0.3

**Figure 3:**
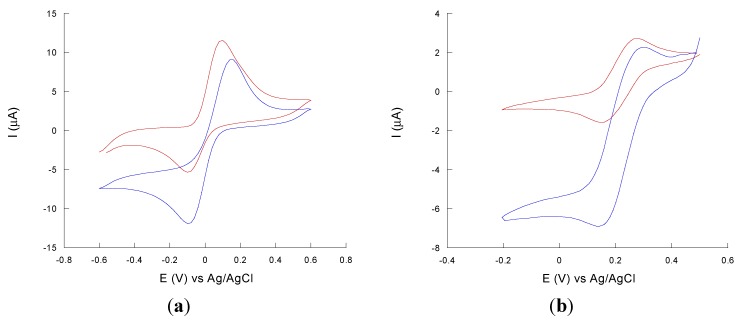
Cyclic voltammograms for 0.5 mM (**a**) 2,2′-Azino-bis(3-ethylbenzothiazoline-6-sulfonic-acid diammonium salt (ABTS) or (**b**) caffeic acid solution onto a MWCNTs-poly(vinylalcohol) (PVA)-AuNPs before (red curve) and after (blue curve) the immobilization of laccase.

A kinetic study was carried out with a non-phenolic compound (*i.e.*, ABTS) and with two phenolic compounds (caffeic acid and catechol). ABTS is often employed as a redox mediator between the enzyme and the electrodes while caffeic acid and catechol are generally considered typical substrates for laccase-based biosensors. The bioelectrochemical behaviour of the laccase modified electrode in the presence of the different redox compounds was characterized by cyclic voltammetry. Cyclic voltammograms at slow scan rates (5 m·Vs^−1^) were recorded in substrates/mediator solution in B-R buffer at pH 5. For all compounds studied, the disappearance of the anodic peak and a large increase in the reduction current is observed, leading to the typical shape of a sigmoidal, catalytic curve (see Figure 3a for ABTS, Figure 3b for caffeic acid), indicating a huge catalytic effect exploited by immobilized laccase.

The shape change arises from the continuous regeneration of the oxidized mediator by the laccase enzyme. The oxidized mediator is then continuously reduced at the electrode surface, thus generating a strong reducing current responsible of the typical S-shape of the catalytic curve. This suggests not only that laccase is efficiently immobilised as witnessed by the large catalytic currents, but also that the immobilization occurs without disrupting the native-like protein structure while preserving the enzyme’s biochemical activity towards its substrates.

Steady-state currents were always observed and kinetic evaluations were solved according to a method based on chronoamperometric measurements recently developed in our laboratories [36].

**Figure 4:**
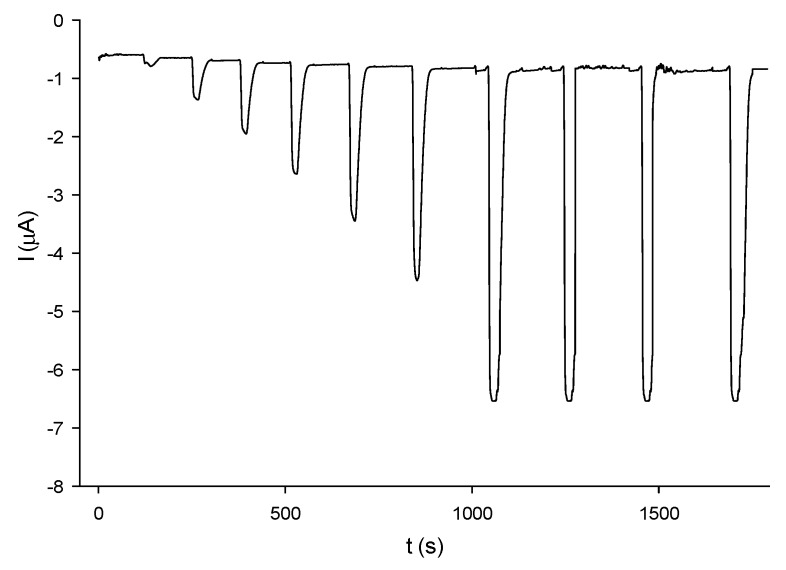
Chronoamperometric current response under flow injection analysis (FIA) conditions to caffeic acid in the range 0.5 μM–1.5 mM, using MWCNTs-PVA-AuNPs-*Trametes versicolor* (TvL) screen-printed electrode. Experimental conditions: 0.1 M B-R buffer pH 5; flow rate: 0.716 mL/min; loop 250 μL, *E* = −0.15 V (*vs*. Ag/AgCl).

For the estimation of the kinetic parameters, the dependence of the catalytic current on the mediator concentration was carried out by chronoamperometry, as shown for example in Figure 4 for caffeic acid. The current intensity is function of both mediator and oxygen concentrations. As the oxygen concentration in water is larger than the *K*_M_ of laccase for oxygen ([O_2_] >> *K*_O2_) where *K* is the corresponding Michaelis constant) [37], the limiting current obtained as the difference between the catalytic current and the diffusion current is related to the substrate concentration according to the Michaelis-Menten equation:

$$I_{\lim}=\frac{I_{\max}[S_{ox}]}{[S_{ox}]+K_{M}^{app}}$$

where [*S*_ox_] is the bulk concentration of the oxidized substrate, *I*_lim_ and *I*_max_ the limiting and maximum current, respectively, and *K*_M_^app^ the apparent Michaelis-Menten constant. *I*_max_ and *K*_M_^app^ values were calculated from Lineweaver-Burk linearization of the above reported equation. The *I*_max_/*K*_M_^app^ ratio gives an estimation of the affinity of the enzyme towards the substrate. A higher value indicates greater effectiveness of the laccase-mediated reduction of oxygen to water. Results of the kinetic study of the laccase biosensor with caffeic acid, catechol and ABTS are presented in Table 3.

**Table 3:** Calculated kinetic parameters of laccase based biosensors for each mediator.

Mediator	Modified Electrode	*I*_max_ (μA)	*K*_M_^app^ (mM)	*I*_max_/*K*_M_^app^ (μA·mM^−1^)	*R*
Caffeic acid	MWCNTs-PVA-AuNPs-TvL	5.98 (± 0.05)	0.027 (± 0.001)	221.48	0.999
	GPH-PVA-AuNPs-TvL	2.89 (± 0.05)	0.053 (± 0.003)	54.23	0.996
Catechol	MWCNTs-PVA-AuNPs-TvL	11.26 (± 0.03)	0.073 (± 0.006)	153.68	0.992
	GPH-PVA-AuNPs-TvL	11.36 (± 0.15)	0.434 (± 0.004)	145.64	0.995
ABTS	MWCNTs-PVA-AuNPs-TvL	13.78 (± 0.24)	0.031 (± 0.002)	444.36	0.996
	GPH-PVA-AuNPs-TvL	4.57 (± 0.16)	0.130 (± 0.010)	35.15	0.996

The comparison of the obtained results show a clear increase of the values of *I*_max_ and a decrease of the values of *K*_M_^app^ for the MWCNTs-PVA-AuNPs-TvL with respect to GPH-PVA-AuNPs-TvL biosensor.

By comparing the calculated *K*_M_^app^ for the different laccase modified electrodes, we observed that it is always lower in the case of MWCNTs with respect to GPH thus suggesting for the first case an increased enzymatic affinity for the substrate. A possible explanation of this behavior is that the more the electrode surface is characterized by a nanosized structure the more it provides a favorable microenvironment for the protein and the protein molecules can reach an orientation which can facilitate the electron transfer from the enzyme to the electrode [38,39].

On the one hand, the increase of *I*_max_ can be explained with the increased electrochemical efficiency of MWCNTs compared to GPH-modified electrodes after the described modifications and already highlighted with mediators in the absence of enzyme. At the same time, the conservation of a native-like structure for the enzyme and the increase of the electroactive surface obtained with a nanostructured transducer could explain also the observed increase of *I*_max_/*K*_M_^app^ which can be considered proportional to the efficiency of the enzymatic turnover. Hence, the improvement of performances results from both increased surface area and an improved electron transfer between the enzyme and the electrode surface.

The analytical performances of the best obtained biosensors (MWCNTs based) towards the different considered compounds are: for caffeic acid the linear range is 1–100 μM with a LOD = 0.5 μM and a sensitivity of 0.051 μA·μM^−1^, for catechol the linear range is 10–200 μM with a LOD = 5 μM and a sensitivity of 0.023 μA·μM^−1^ and for ABTS the linear range is 0.5–75 μM with a LOD = 0.2 μM and a sensitivity of 0.057 μA·μM^−1^. These values are in good agreement with and in some case slightly better than those reported in literature for similar laccase-based biosensors [1,22,23,24], confirming the usefulness of the approach reported herein.

## 3. Experimental Section

### 3.1. Chemicals

Fungal laccase from *Trametes versicolor* (TvL) (E.C. 1.10.3.2, activity: 30.6 U/mg) was purchased from Fluka (Bucks, Switzerland). The laccase enzyme was kept stored at −18 °C.

Potassium ferricyanide, ferrocene monocarboxylic acid (FMCA), 2,2′-Azino-bis(3-ethylbenzothiazoline-6-sulfonic-acid diammonium salt (ABTS), 1,2-dihydroxybenzene (catechol), and colloidal gold nanoparticles (AuNPs) (diameter 5 nm and 10 nm) were purchased from Sigma-Aldrich (St. Louis, MO, USA). Solutions of mediators were prepared in 0.1 M phosphate buffer pH 7.0 and in 0.1 M Britton-Robinson (B-R) buffer pH 5, immediately before use.

The polymeric film employed for protein entrapping was poly(vinyl alcohol), *N*-methyl-4(4′-formylstyryl)pyridinium methosulfate acetal (PVA-SbQ), supplied by Polysciences Inc. (Warrington, PA, USA). In the PVA-SbQ encapsulation the photo-cross-linking of the styrylpyridinium groups of the PVA-SbQ creates a network for biomolecule entrapping.

Other chemicals were all of analytical grade. All solutions used throughout experiments were prepared with high-purity deionized water obtained from a filtration station Millipore (Molsheim, France).

### 3.2. Apparatus

Amperometric experiments were performed using a DropSens potentiostat μStat200 (Oviedo, Spain) in a 10 mL glass cell with a conventional three electrodes configuration. The working electrode (WE), the pseudo-reference electrode (RE), and the counter electrode (CE) were assembled as screen-printed electrodes. The WE was constituted by either graphene (GPH) or multi walled carbon nanotubes (MWCNTs) (DropSens). The surface diameter of the WE was 2 mm. The RE was an Ag/AgCl pseudo-reference electrode and the CE was made of graphite.

Flow experiments were carried out using a Gilson Minipuls-3 peristaltic pump and a microliter flow cell for screen-printed electrodes (DropSens).

### 3.3. Electrode Modification with AuNPs

The GPH and the MWCNTs screen-printed electrodes were modified by depositing onto the electrode surface 2 μL of a colloidal solution of AuNPs of either 5 or 10 nm diameter at the following concentrations: ≈ 7 × 10^−4^ mM, ≈ 1 × 10^−3^ mM, ≈ 1.4 × 10^−3^ mM. Successively 4 μL of the PVA-SbQ photopolymer were spread onto the electrode surfaces. Then the electrodes were left under a UV-VIS lamp for 20 min to allow the entrapping of the AuNPs onto the electrode surface.

### 3.4. GPH-PVA-AuNPs-TvL and MWCNTs-PVA-AuNPs-TvL Biosensor Preparation

In order to prepare the laccase biosensor, the GPH and MWCNTs screen-printed electrodes were modified by depositing onto the electrode surface in sequence 2 μL of a colloidal solution of AuNPs 1.4 × 10^−3^ μM, 4 μL of the photopolymer PVA-SbQ and 3 μL of a solution containing 0.306 U/mL of TvL. After the deposition, the electrodes were left under a UV-VIS lamp for 20 min to allow the entrapping of both AuNPs and the enzyme onto the electrode surface.

### 3.5. Electrochemical Measurements

Cyclic voltammetry experiments were performed in a 10 mL cell at a scan rate of 5 m·Vs^−1^ in phosphate buffer 0.1 M, pH 7 and 0.1 M B-R buffer pH 5.

Flow experiments were carried out at a fixed potential of −0.15 V with a flow rate of 4 μL·s^−1^. The carrier buffer was 0.1 M B-R pH 5 and aliquots of 100 μL of ABTS standard solutions at different concentrations in the same buffer were used to obtain the calibration plot.

All the values reported are the average of 5 measurements.

## 4. Conclusions

This experimental research involved the construction and characterization of a nanostructured electrode material for the development of an electrochemical biosensor that exploits the synergistic benefits of nanomaterials based on carbon (multi-walled nanotubes and graphene) and gold nanoparticles.

The electrochemical behavior of the electrode surface, before and after the different modification procedures, was analyzed through cyclic voltammetry experiments in the presence of different redox mediators and an increase in performance of the electrochemical biosensor according to different procedures for modifying the electrode surface, thanks to the previously identified conductive features and catalytic properties of nanomaterials, was observed.

Basing on these results, the expected benefits and the good potential of the modification of the electrode surface with nanomaterials for the development of electrochemical biosensors has been confirmed.

The strategy proposed for the modification of the electrode in this research work therefore opens up significant future applications for biosensors in clinical-medical, pharmaceutical, environmental and food application fields.
